# Bullous Erythema Multiforme Secondary to Trimethoprim-Sulfamethoxazole Use, Treated With Cyclosporine in a 91-Year-Old Male

**DOI:** 10.7759/cureus.18239

**Published:** 2021-09-24

**Authors:** Simon Kashfi, Ashley A Radparvar, Yoseli Ventura, Sapna Sharma, Shorabh Sharma

**Affiliations:** 1 Internal Medicine, CUNY School of Medicine, New York, USA; 2 Internal Medicine, Albert Einstein College of Medicine, New York, USA; 3 Internal Medicine, SBH Health System, New York, USA; 4 Internal Medicine, Mahatma Gandhi Mission Institute of Health Sciences, Navi Mumbai, IND

**Keywords:** toxic epidermal necrolysis (ten), fixed drug eruption, steven johnson syndrome, cyclosporine, erythema multiforme

## Abstract

Erythema multiforme is an acute, immune-mediated, mucocutaneous condition in which 90% of cases are triggered by infection. The second most common cause is drug-induced. It classically presents with itchy, burning targetoid lesions on the skin and mucous membranes. The lesions may be mistaken for other conditions, and thus, rapid and correct diagnosis is crucial. It is most often treated with corticosteroids, though non-responders or those with weakened immune systems may require immunomodulatory therapy. We present the case of a 91-year-old male who developed bullous erythema multiforme after treatment with trimethoprim-sulfamethoxazole who was successfully treated with cyclosporine.

## Introduction

Erythema multiforme (EM) is an acute, immune-mediated, mucocutaneous condition in which 90% of cases are triggered by infection, namely the herpes simplex virus (HSV) [1]. It can also occur in the setting of medication use. It was first described in 1860 by Ferdinand von Hebra, and cases with mucosal involvement have been reported since 1876 [2]. The exact incidence of EM is unknown, but it is thought to be between 0.01% and 1% [1,3]. It most often occurs in younger adults, with a slight female predominance [3]. EM can be classified into minor and major forms; EM minor lacks mucosal involvement [1]. Lesions of EM are typically targetoid plaques, with a dark center surrounded by two lighter zones. It is a self-limiting condition; the lesions appear over several days and resolve in 1-2 weeks, though the resolution may take longer in EM major. It is diagnosed clinically, with help from histopathology. Treatment for EM involves managing or removing the underlying cause, and starting either topical or oral glucocorticoids depending on the condition’s severity [4]. Immunomodulatory therapies have been explored as well, though no large-scale clinical trials have been performed [2-5]. We present the case of a 91-year-old male who developed bullous EM after treatment with trimethoprim-sulfamethoxazole (TMP-SFX) for a urinary tract infection, who was successfully treated with cyclosporine.

## Case presentation

The patient is a 91-year-old male with a history of HIV (on highly active antiretroviral therapy, CD4 count 701 μL, viral load undetected), Parkinson’s disease, benign prostatic hyperplasia, and chronic obstructive pulmonary disease on home oxygen who presented to the emergency department with a rash for one week. The rash initially started as a nonpruritic, erythematous, and tender spot on his anterior right lower leg, but then spread to both arms and then his lower lip. The rash did not follow a dermatomal distribution. The patient had begun Trimethoprim/Sulfamethoxazole (TMP-SFX) for a urinary tract infection two days before the onset of the rash. It was unknown if this was the patient’s first exposure to this drug.

**Figure 1:**
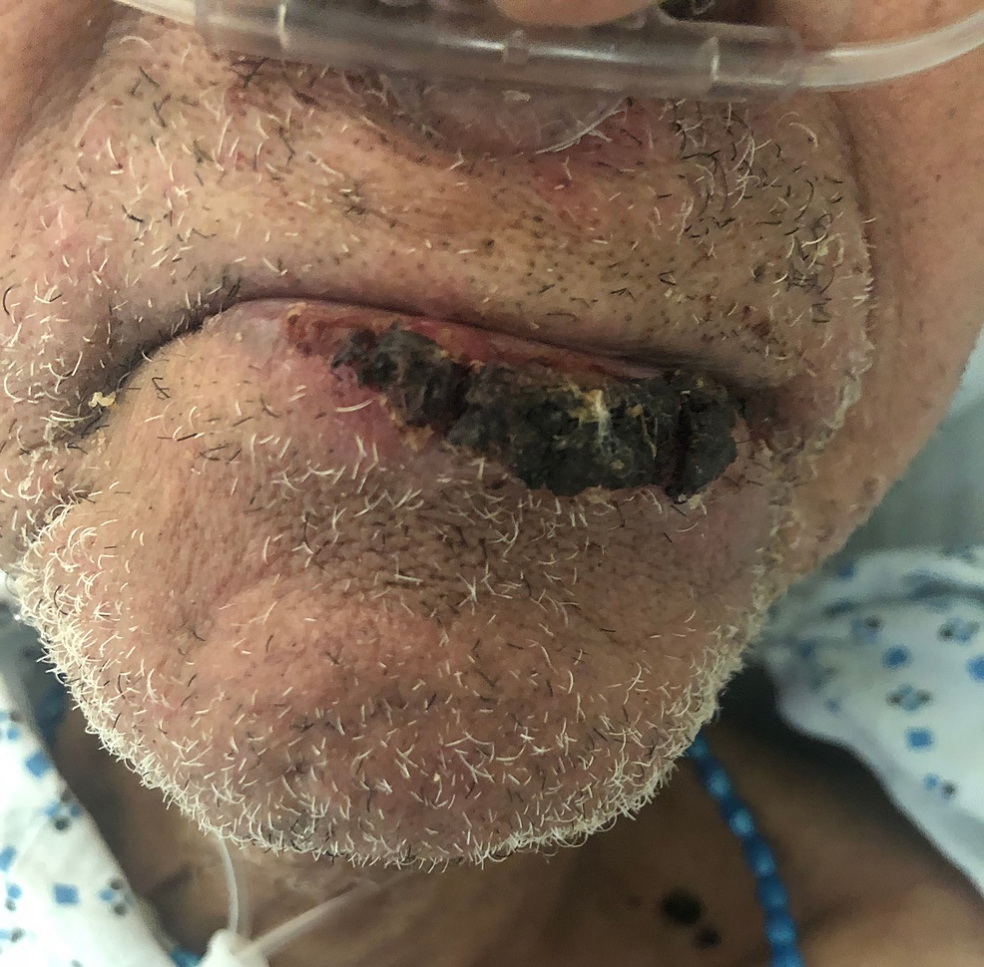
Encrusted lesion of the lower lip

On exam, the patient was afebrile, with a blood pressure of 108/74 mmHg, heart rate of 81 beats per minute, respiratory rate of 17 breaths per minute and normal oxygen saturation on room air. The lesions were targetoid plaques with dusky centers, ranging from 1-4cm and involving the bilateral arms and legs. There were about 15 or so acrally distributed lesions in total. They spared the palms and soles, and at this point, none had bullous morphology. There was also involvement of the oral mucosa and lower lip, which was swollen and crusted (Figure 1). Nikolsky’s sign was negative. Punch biopsy and frozen section were performed from a lesion on the shoulder. Ophthalmology was consulted to rule out eye involvement, as was the local burn center for possible transfer if the patient had Steven Johnson’s Syndrome (SJS). The patient had no evidence of eye involvement, and the burn center did not accept the patient because of a lack of histological confirmation of SJS. Laboratory evaluation was unremarkable, and HSV serology was not obtained.

**Figure 2:**
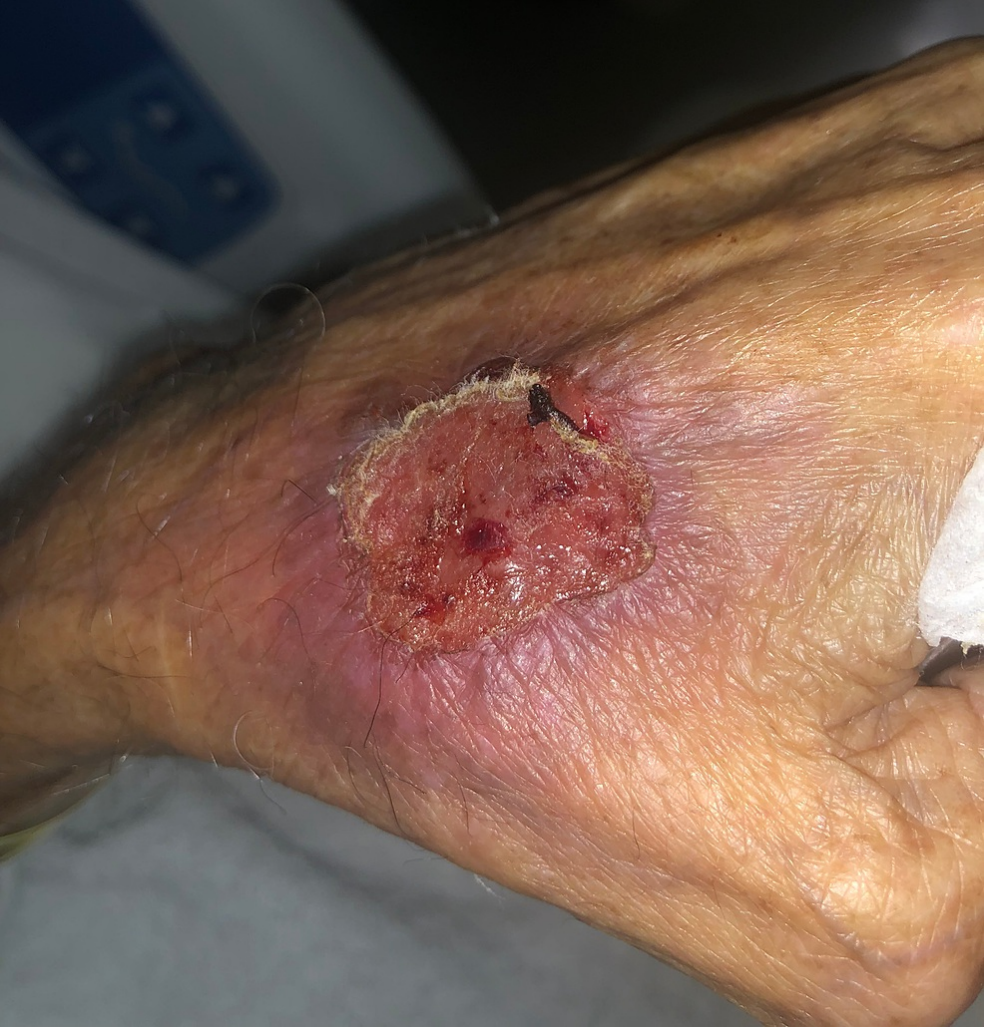
Healing targetoid lesion on the patient's dorsal hand

**Figure 3:**
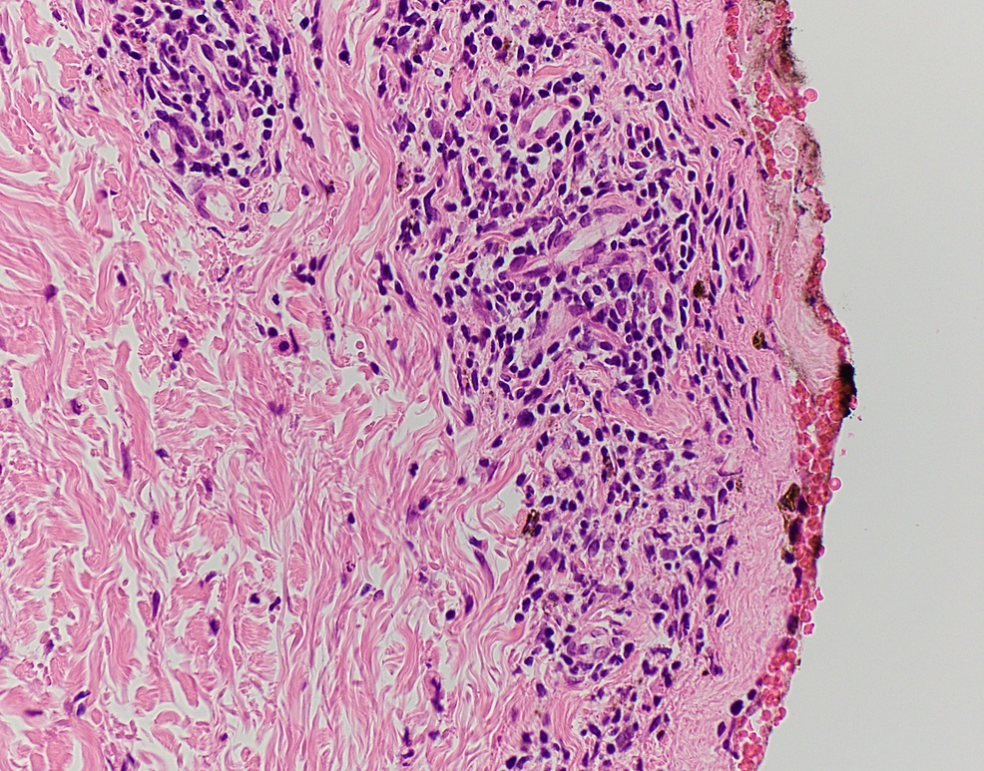
Skin biopsy showing absence of the epidermis and an underlying inflammatory, lymphocytic, non-vacuolar lichenoid dermatitis consistent with bullous erythema multiforme.

Dermatology was consulted and recommended to start IV cyclosporine at a dose of 3mg/kg while waiting for the biopsy results. At this time, the differential diagnosis was erythema multiforme vs SJS. TMP-SFX was discontinued. The next day, the lesions were described as targetoid with sloughing of the center on an erythematous base. There was no progression or worsening of the lesions. On the third day, the patient’s lesions greatly improved (Figure 2). Most notably, swelling of the lower lip had decreased. The pathology report showed the absence of epidermis and underlying lichenoid dermatitis consistent with bullous erythema multiforme (Figure 3). The patient was discharged on an oral cyclosporine taper for 20 days, with outpatient follow-up. His lesions had nearly cleared by discharge.

## Discussion

The differential diagnosis of EM is large [1,4]. Most relevant to our case are SJS and fixed drug eruption (FDE). SJS is a mucocutaneous condition that most often occurs as a response to new medications [2]. It is similar to EM in that it is also characterized by targetoid lesions on the skin and mucous membranes. However, the lesions are macular in SJS and papular in EM, though histologically they are difficult to distinguish [1]. EM lesions feature liquefactive degeneration of the basal epidermal cells, necrotic keratinocytes, and exocytosis of lymphocytes. There can be lymphohistiocytic infiltrates at the dermo-epidermal junction. This is known as interface dermatitis, which our patient had [1,2]. Additionally, the lesions in SJS are more often found on the trunk, while in EM they are most often on the acral extremities. The two were once thought to be on a continuum, though they are now considered different entities. Rather, SJS is on a continuum with toxic epidermal necrolysis (TEN). SJS covers 10% of the body surface area, and TEN 30% [6,7]. These patients require burn units for treatment management. 

FDE is a cutaneous reaction that occurs in response to a medication. It accounts for 10% of all cutaneous drug reactions, and a rarer variant of FDE is known as generalized FDE [8]. Lesions in FDE typically occur in the same area of the skin each time a drug is taken. On skin exam, FDE is quite similar to EM, but FDE will usually have fewer lesions than EM. On histopathology, FDE also has interface dermatitis, though with deeper extension of the infiltrate and fewer neutrophils [1,4,8].

Our patient’s condition was triggered by TMP-SFX use. Sulfonamide drugs are a cause of hypersensitivity, which can occur in up to 7% of patients [9]. In patients with cutaneous toxicity secondary to antibiotics, TMP-SFX accounts for 15.3% of cases [10]. Sulfonamides cause adverse reactions through a non-IgE mediated hypersensitivity reaction. Current mechanisms for toxicity included a hapten-mediated reaction that ultimately leads to antigen presentation and subsequent immune-mediated hypersensitivity. The most common cutaneous reaction to sulfonamides is maculopapular eruptions, which accounts for as much as 36% of cutaneous reactions [10]. Other cutaneous reactions due to sulfonamide use include SJS, TEN, and drug reaction with eosinophilia and systemic symptoms syndrome. Of note, Firoz et al. found that the most common culprit of TEN was TMP-SMX, accounting for as many as 36.6% of reactions [11]. Extracutaneous reactions to sulfonamides include liver and renal injury along with gastrointestinal sequelae and hematologic abnormalities [12].

As mentioned, 90% of cases of EM are caused by infection, with HSV-1 being the most common culprit. The second most common pathogen is *Mycoplasma pneumoniae*, and this is especially true in children and young adults [1,2]. Drug-induced EM accounts for <10% of cases, and the drugs with the highest risk are antiepileptics, antibacterial sulfonamides, allopurinol, and oxicam NSAIDS [2]. Moderate risk drugs are phenylacetic NSAIDS and several classes of antibiotics - cephalosporins, quinolones, macrolides, and tetracyclines. Drugs with no increased risk are antihypertensives, including sulfonamide diuretics, and propionic acid NSAIDS. Important here is the patient’s haplotype, which can predispose patients of certain ethnicities to EM depending on the exposure [1]. Our patient’s haplotype was not assessed, and it is not routinely done so.

EM can be divided into minor and major forms. EM major has mucosal involvement, while EM minor does not. Mucosal lesions in EM occur 25-60% of the time, and it is rare to have mucosal lesions without skin lesions. The lesions themselves can be divided into typical and atypical forms [1-4]. Typical EM lesions are called target or iris lesions because they have three concentric rings of different colors. There is a dark center surrounded by a light pink zone, both of which are encircled by a red zone. Atypical lesions can also occur. These feature only two rings of color and/or a poorly defined border. Our patient had EM major with typical target lesions, though unfortunately, we were unable to take pictures at the time of presentation. Though the disease course is self-limiting, it can cause significant debility. The main cause of morbidity in patients with EM major is decreased oral intake. Mucosal lesions are quite painful, and patients often require intravenous fluids and electrolytes [4]. Additionally, clinicians should have a low threshold for observation for admission because EM is so difficult to distinguish from SJS. The skin lesions itch and burn, and cause swelling of the hands and feet. At the end of the illness course, the lesions usually resolve without complication, though post-inflammatory skin hyperpigmentation can occur for several months [1,4]. 

The pathophysiology of EM differs by the underlying cause. For virus-induced EM, IFN-γ is upregulated when virus fragments are transported to the epithelium, causing T cells to accumulate. IFN-γ then stimulates the production of cytokines that attract T and NK cells to the skin surface. This is considered a delayed-type hypersensitivity reaction. In drug-induced EM, metabolites of the drug cause keratinocyte apoptosis, causing the release of TNF-α [3]. 

A treatment algorithm for EM has been described [4]. The first step is to manage the inciting event. This means stopping medication or treating an HSV or *Mycoplasma pneumoniae *infection. Second, note that the preferred treatment is a steroid, and the use of topical vs oral vs IV depends on the severity of the condition and is at the discretion of the treating physician. Severe cases can be treated with a month-long steroid taper. Of interest, our patient, a 91-year-old male with HIV, was treated with cyclosporine, an IL-2 inhibitor and biologic agent [13]. Cyclosporine works by binding to cyclophilin, and this complex inhibits calcineurin, which normally activates T cells. Thus, cyclosporine inhibits T cells and exerts an immunosuppressive effect. Cyclosporine has been reported to be successful in treating a case of recurrent EM [5], a rare form of EM that, as its name suggests, is chronic and associated with HSV [1]. It has also been used in a case of severe bullous EM [14], and generalized bullous FDE [8,15]. Its use in generalized bullous FDE has been supported by studies that show a rapid cessation of blistering and healing of skin [15], while providing a steroid-sparing effect. There are no studies comparing cyclosporine to steroids for EM. Our immunocompromised and elderly patient had an excellent clinical response to cyclosporine and avoided the adverse effects of corticosteroids.

## Conclusions

The differential diagnosis for a mucocutaneous reaction to a drug is large. EM, SJS, and TEN should be considered in these patients because of dangerous sequelae. The diagnosis of EM is supported with a history of HSV infection or the introduction of a new drug. Typical target lesions and histopathology are used to support and confirm the diagnosis. EM is most commonly treated with steroids. The modality of treatment can be dependent on the severity of the condition. Our case shows that first-line treatment should be tailored to the patient's specific needs; the 91-year-old man with HIV was treated with cyclosporine, a more aggressive option.
